# Cemented or uncemented hemiarthroplasty for the treatment of femoral neck fractures? A Finnish database study of 25,174 patients

**DOI:** 10.3109/17453674.2013.878827

**Published:** 2014-02-25

**Authors:** Tero Yli-Kyyny, Reijo Sund, Mikko Heinänen, Petri Venesmaa, Heikki Kröger

**Affiliations:** ^1^Department of Orthopedics,Traumatology and Hand Surgery, Kuopio University Hospital and University of Eastern Finland,Kuopio; ^2^National Institute for Health and Welfare,Helsinki, Finland and University of Eastern Finland,Kuopio; ^3^Department of Orthopedics and Traumatology,Töölö Hospital, Helsinki University Central Hospital, Helsinki,Finland.

## Abstract

**Background and purpose:**

Cemented hemiarthroplasty is preferred in treating displaced fractures of the femoral neck in the elderly. The cementing process may cause a fat embolism, leading to serious complications or death. In this study, we wanted to determine whether use of uncemented hemiarthroplasty (HA) would lead to reduced mortality and whether there are differences in the complications associated with these different types of arthroplasty.

**Patients and methods:**

From the PERFECT database, which combines information from various treatment registries, we identified 25,174 patients who were treated with hemiarthroplasty for a femoral neck fracture in the years 1999–2009. The primary outcome was mortality. Secondary outcomes were reoperations, complications, re-admissions, and treatment times.

**Results:**

Mortality was lower in the first postoperative days when uncemented HA was used. At 1 week, there was no significant difference in mortality (3.9% for cemented HA and 3.4% for uncemented HA; p = 0.09). This was also true after one year (26% for cemented HA and 27% for uncemented HA; p = 0.1). In patients treated with uncemented HA, there were significantly more mechanical complications (3.7% vs. 2.8%; p < 0.001), hip re-arthroplasties (1.7% vs. 0.95; p < 0.001), and femoral fracture operations (1.2% vs. 0.52%; p < 0.001) during the first 90 days after hip fracture surgery.

**Interpretation:**

From registry data, mortality appears to be similar for cemented and uncemented HA. However, uncemented HA is associated with more frequent mechanical complications and reoperations.

Displaced fractures of the femoral neck are being increasingly treated with arthroplasty instead of osteosynthesis (Rogmark et al. 2010). Hemiarthroplasty (HA) is used in most patients (Bhandari et al. 2005). The operation can be performed using either cemented or uncemented femoral components. Cemented components have been preferred, since they have been associated in meta-analyses with less postoperative pain and better mobility after surgery (Parker et al. 2010). However, these studies have mostly compared relatively outdated non-modular types of hemiarthroplasty.

After the introduction of modular hemiarthroplasty to hip fracture surgery in recent years, a number of prospective trials comparing cemented and uncemented hemiarthroplasty have been published, with very similar results for both (Figved et al. 2009, DeAngelis et al. 2012, Taylor et al. 2012). However, in a recent registry study comparing (mostly modular) cemented and uncemented hemiarthroplasty, more reoperations were detected in patients treated with uncemented hemiarthroplasty (Leonardsson et al. 2012). One explanation for this discrepancy may be the relatively small sample size and incomplete follow-up associated with prospective studies (Talsnes et al. 2013).

We studied mortality and results after hemiarthroplasty using Finnish registry-based data.

## Patients and methods

Patients with a first femoral neck fracture who were operated with hemiarthroplasty of the hip in Finland and admitted to a surgical ward between January 1, 1999 and December 31, 2009 were identified from the Finnish Hospital Discharge Register (FHDR) using the tenth revision of the International Classification of Diseases (ICD-10) diagnosis code S72.0 and the Finnish version of NOMESCO Classification Procedural Codes NFB10 (uncemented hemiarthroplasty) or NFB20 (cemented hemiarthroplasty).

Data on comorbidities, on the use of residential care, and on deaths in this population were extracted from the Finnish Health Care Register (i.e. the reimbursement register (prescription database) of the Social Insurance Institution), using the unique personal identification number of each patient. Records in these registers include data such as: patient ID number, provider ID number(s), age, sex, area codes, diagnosis and operation codes, and also dates of admission, operation, and discharge (or death). The information from these registers has been gathered in the PERFECT (PERFormance, Efficiency, and Costs of Treatment Episodes) database. The Finnish registry data from the PERFECT database concerning hip fracture patients have been compared to prospective audit data (Sund et al. 2007). The completeness of the registry data was good; the positive agreement between audit data and registry data was 94.9%. Also, the accuracy of easily measurable variables in the registry data was at least 95%.

The validity of the individual registries mentioned above has also been studied. The Finnish Hospital Discharge Register data have been compared to external audit data in 32 studies (Sund 2012). The coverage and positive predictive values for injury diagnoses have been over 90% in those studies. The prescription database data have been found to be in high concordance with self-reported medication (Haukka et al. 2007).

Reasons for death were extracted from the national Causes of Death Statistics. In Finland, injury-related deaths lead to a forensic autopsy in over 85% of cases, which is a higher rate than in most other countries (Lunetta et al. 2007). The validity of the Finnish mortality statistics has also been studied and has been found to be reliable (Lahti and Penttilä 2003, Pajunen et al. 2005).

The primary outcome used in this study was total mortality. Secondary outcomes included new hip operations (procedure codes NFB*, NFC*, NFH*, NFJ*, NFS*, NFU*, NFW*, NFX*, and other NF*) and complications related to surgery or implant (diagnostic codes T84.0 + T84.1 (mechanical complications), T81.4 + T84.5 (infectious complications), T93.1 (late effects), S73.0 (hip luxations), and S72.1–S72.4 (femoral fractures distal to the femoral neck)) as well as medical complications (I21 (acute myocardial infarction), I25 (ischemic heart disease), I26 (pulmonary embolism), I50 (heart failure), and I63 (stroke) within 90 days since the index procedure). The procedural codes used in this study are shown in Table 1.

**Table 1.** Procedural codes (NOMESCO classification) used in this study

Code	Procedure
NF	Hip and thigh
NFB00–99	Primary prosthetic replacement of hip joint
NFB10	Primary hemiarthroplasty of hip joint not using cement
NFB20	Primary hemiarthroplasty of hip joint using cement
NFC00–99	Secondary prosthetic replacement of hip joint
NFH00–99	Miscellaneous operations on hip joint
NFJ00–99	Fracture surgery on hip joint
NFS00–99	Operations for infected tendons, joints, and bones of hip and thigh
NFU00–99	Removal of implants and external fixation devices from hip and femur
NFW00–99	Reoperations on hip or thigh

### Study population

**Figure 1.**
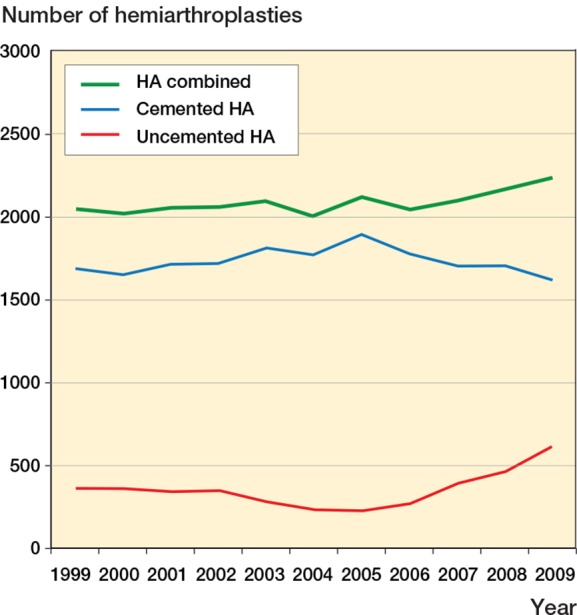
Annual numbers of cemented and uncemented hemiarthroplasties in Finland during the study period.

During the study period, 25,174 patients were treated with hemiarthroplasty for a femoral neck fracture in Finland. The use of cemented hemiarthroplasty remained constant in Finland during the study period. The use of uncemented hemiarthroplasty has increased in recent years, leading to a slightly increased total use of hemiarthroplasty (Figure 1). Background information about the study patients is given in Table 2.

**Table 2.** Background information on the patients, including duration of treatment, mortality, and cost of treatment. These results have not been adjusted via propensity score weighting

	Uncemented HA	Cemented HA	p-value for difference (unadjusted)
No. of patients	4,492	20,682	
Background characteristics			
Mean age	81	81	0.002
Men, %	26.2	26.2	0.5
Admitted from long-term care, %	12.9	11.9	0.04
Patients using calcium and vitamin D, %	20.5	18.3	< 0.001
Patients using antiosteoporotic agents, %	17.3	17.8	0.2
Heart disease, %	46.5	39.2	< 0.001
Alcoholism, %	2.7	2.7	0.4
Cancer, %	12.7	13.5	0.08
Chronic obstructive pulmonary disease, %	14.3	13.2	0.02
Dementia, %	23.8	20.4	< 0.001
Depression, %	25.6	24.9	0.2
Parkinson’s disease, %	6.2	6.2	0.4
Mental disorders, %	14.1	13.4	0.1
Rheumatoid arthritis, %	6.7	6.2	0.1
Cerebrovascular disease, %	19.3	16.7	< 0.001
Treatment, outcomes and costs			
30-day mortality, %	8.7	8.6	0.4
Home at day 120, %	53.5	56.9	< 0.001
120-day mortality, %	18.0	17.1	0.07
Home at day 365, %	49.0	53.0	< 0.001
365-day mortality, %	27.4	25.4	0.003
The average duration of first treatment			
episode in hospital, days	6.6	7.4	< 0.001
Mean costs until discharge home, in €	10,990	12,050	< 0.001

The exact type of hemiarthroplasty (implant manufacturer and model) is not registered in the PERFECT database. We therefore sent an e-mail survey to Finnish hospitals performing hemiarthroplasties (May to October 2012). Of the 14 hospitals contacted, accounting for over 70% of hemiarthroplasties in Finland, none had used non-modular uncemented stems after 1999. Cemented non-modular hemiarthroplasty had been used up until 2005 in 4 of the hospitals contacted. On the basis of this survey, we were able to determine that use of non-modular uncemented hemiarthroplasty has been infrequent in Finland during the study period.

### Statistics

Mortality between the cemented and uncemented groups was examined using logistic regression analysis. The analysis was repeated for 365 outcomes that each described the status of the patient (alive/dead) on a certain day after the operation. In order to reduce the effects of confounding in this observational study, differences in distributions of observed covariates between the groups were adjusted using propensity score weighting (Austin 2011). Propensity scores, i.e. the probabilities of treatment assignment conditional on observed baseline characteristics, were calculated using a generalized boosted regression model (McCaffrey et al. 2013). In the model, treatment assignment (cemented/uncemented) was the dependent variable and all observed background variables listed in Table 2 were independent variables, as the aim was to balance all observed covariates between the groups. Secondary outcomes were analyzed using Cox’s regression model, also adjusted via propensity score weighting. We assumed non-informative censoring and follow-up was until outcome event or censoring due to death or last day of the observation period, whichever occurred first. Proportional hazards assumption was tested using weighted Schoenfeld residuals. We also performed sensitivity analyses using proportional hazards model for competing risks proposed by Fine and Gray for secondary outcomes, as death can be considered as a competing risk for those events. As the results were almost identical, we report only the results from Cox regression. Data preprocessing and analyses were performed with the R software package 2.15.3 and extension packages muste 0.5.39, twang 1.3-11, and cmprsk 2.2-6.

### Ethics

The ethical committee of the Finnish National Institute for Health and Welfare (THL) approved the study (THL TuET §138/2010).

## Results

**Figure 2.**
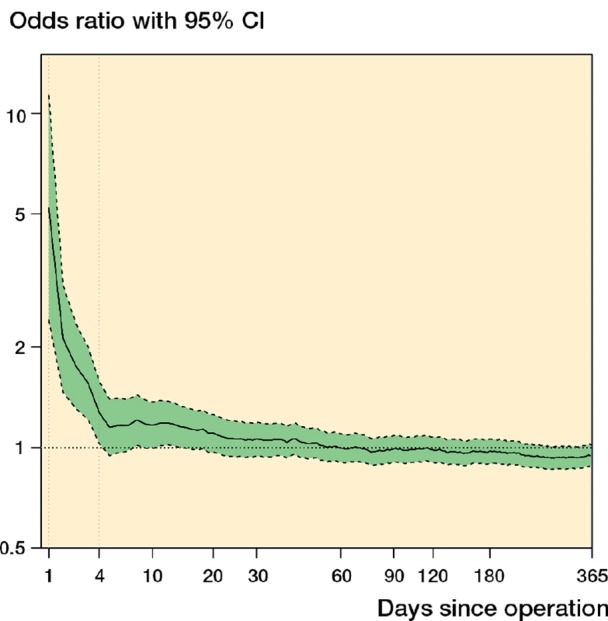
The relative and cumulative risk of death in patients receiving a cemented hemiarthroplasty compared to patients receiving an uncemented hemiarthroplasty. Mortality was higher in the cemented group until day 4. From day 5 onward, no statistically significant difference in mortality was found.

The initial mortality after surgery with cemented HA was higher than with uncemented HA (Figure 2). At day 1 postoperatively, the mortality for cemented HA was 1.49% and that for uncemented HA was 0.73% (OR = 2.12; p < 0.001). The difference in mortality at day 4 was still statistically significant (2.90% for cemented HA and 2.36% for uncemented HA; OR = 1.27; p = 0.03). At 5 days, the difference was no longer statistically significant. At 1 week, the mortality for cemented HA was 3.94% and for uncemented HA it was 3.41% (OR = 1.16; p = 0.1). During the follow-up, there was a trend of lower mortality in patients who received cemented HA, but it did not reach statistical significance at one year (25.6% vs. 26.7%; p = 0.2).

We were also interested in whether the fat embolism would be cited more often as one of the causes of death in patients who received cemented HA. In the cemented group, a fat embolism was cited as one of the causes (primary or contributory) of death in 14% of cases and in the uncemented group this was present in 9.0% of cases.

We also studied mortality in hospitals that had performed more than 100 cemented HAs and more than 100 uncemented HAs during the study period. The mortality at 1 week was 4.4% for cemented HA and 3.5% for uncemented HA (p = 0.06). In all, the mortality rates did not differ statistically significantly from the mortality of the study group as whole.

Patients treated with cemented HA tended to have less morbidity, and were eventually discharged home more often than patients treated with uncemented HA. The cost of treatment was higher in patients treated with cemented HA (Table 2).

Complications of hip fracture treatment with HA were studied by comparing the occurrence of certain diagnostic codes and procedural codes for operated patients during the first 90 days after hip fracture surgery (Table 3). There were more mechanical complications and more new surgical procedures in patients treated with uncemented HA.

**Table 3.** The occurrence of certain diagnostic codes and new operations on the hip joint in patients within 90 days of the index procedure

	Uncemented HA (%)	Cemented HA (%)	HR (95% CI)	p-value for difference (unadjusted)
Ischemic heart disease (acute)	2.41	2.16	0.90 (0.73–1.11)	0.3
Ischemic heart disease (chronic)	8.40	7.86	0.93 (0.83–1.04)	0.2
Pulmonary embolism	0.94	0.86	0.92 (0.66–1.28)	0.6
Heart insufficiency	4.21	4.71	1.12 (0.96–1.30)	0.1
Cerebrovascular disturbances	2.58	2.79	1.08 (0.89–1.32)	0.4
New femoral fractures	2.85	1.63	0.57 (0.46–0.70)	< 0.001
Infectious complications	2.30	1.89	0.82 (0.66–1.02)	0.08
Mechanical complications	3.72	2.77	0.74 (0.62–0.88)	< 0.001
Re-arthroplasties of hip (NFC00–99)	1.66	0.95	0.57 (0.43–0.74)	< 0.001
Fracture surgeries on femur (NFJ00–99)	1.19	0.52	0.44 (0.32–0.61)	< 0.001
Operations for infection (NFS00–99)	0.42	0.27	0.65 (0.38–1.10)	0.1
Implant removals (NF00–99)	1.29	0.78	0.60 (0.44–0.82)	0.001
Reoperations on hip or thigh (NFW00–99)	0.76	0.50	0.66 (0.44–0.97)	0.03

The number of new treatment periods for various medical complications (e.g. ischemic heart attacks and cerebrovascular disturbances) during the first 90 days was similar between the groups (Table 3).

## Discussion

The use of hemiarthroplasty for treatment of femoral neck fractures has remained stable in Finland during the years 1999–2009. Uncemented hemiarthroplasty has gained more popularity during the most recent years, possibly related to the introduction of newer prosthetic implant models to hip fracture surgery. The use of osteosynthesis with screws for femoral neck fractures has been relatively infrequent in Finland. This was reflected in the present study, as we did not observe the increase in hemiarthroplasties noted in Sweden (Rogmark et al. 2010), where osteosynthesis has traditionally been a more popular choice for femoral neck fractures.

Cemented hemiarthroplasty has been preferred over uncemented hemiarthroplasty because of less postoperative pain and better mobility (Parker et al. 2010). Most studies comparing cemented and uncemented hemiarthroplasty have compared relatively outdated non-modular stems. It appears that the use of monoblock prostheses is diminishing, making these studies potentially outdated (Rogmark et al. 2012). There have been a few prospective, randomized studies comparing modular, contemporary hemiarthroplasty with and without bone cement. In these studies, uncemented, modular hemiarthroplasty has given equivalent results to cemented hemiarthroplasty in terms of functional outcomes, complications, and mortality (Figved et al. 2009, DeAngelis et al. 2012, Taylor et al. 2012, Talsnes et al. 2013).

In a recent Swedish registry study, uncemented hemiarthroplasty was found to be associated with more reoperations than cemented hemiarthroplasty (Leonardsson et al. 2012). We detected the same in our study. We found more hip re-arthroplasties, fracture surgeries on the femur, implant removals, mechanical complications, and reoperations on the hip in patients treated with an uncemented hemiarthroplasty. Our and Leonardsson’s findings are of interest since they contradict the results of prospective trials (Figved et al. 2009, DeAngelis et al. 2012, Taylor et al. 2012). We believe that this may reflect the difficulties faced when the results of prospective trials are applied to clinical practice. Prospective, randomized trials usually have high internal validity, but as a result a portion of the population of interest is excluded from the study. This may lead to a situation where clinical results are different from the results of randomized trials.

Early postoperative mortality after cemented hemiarthroplasty was higher in our study. This could be a consequence of “bone cement implantation syndrome” (Donaldson et al. 2009), where fat and bone marrow cause emboli in pulmonary arteries, as fat embolism was more often detected in deceased patients who had received cemented hemiarthroplasty. However, the difference in mortality was reversed in the follow-up, and a trend of lower mortality was seen in patients treated with cemented hemiarthroplasty. In this respect, our results reflect the trends seen in Australian registry data (Costain et al. 2011). In contrast to their results, though, we did not see a statistically significant increase in 1-year mortality associated with uncemented hemiarthroplasty. It should be noted also that in a recent British database study, no increase in perioperative mortality was detected with the use of cement (Costa et al. 2011). In light of these slightly contrasting results, it appears that the fixation method itself has little effect on mortality, at least beyond the first postoperative week.

Treatment times and treatment costs were higher in patients treated with cemented hemiarthroplasty. It should be noted, however, that these results were obtained without adjustment through propensity score weighting. Consequently, these results may have been caused by other differences between the study groups.

The limitations of the present study include the general drawbacks of registry-based studies, i.e. their retrospective nature and reliance on diagnostic codes used during normal clinical practice. The validity of the Finnish PERFECT database has been shown to be good, though (Sund et al. 2011). Another limitation was the lack of information about the type of implant used for surgery. We conducted an e-mail survey and contacted hospitals accounting for over 70% of hemiarthroplasties in Finland. It seems that the use of uncemented, non-modular hemiarthroplasty was very limited in Finland during the study period, which is similar to the situation in Sweden (Rogmark et al. 2012). Despite these limitations, we believe that registry-based studies have an important role in hip fracture research and should be encouraged. We believe that an optimal hip fracture registry would include operative details including implant type and information about possible reoperations. However, the data gathered should also include information about postoperative treatment, rehabilitation, and medical complications. In this regard, we think that a modern arthroplasty registry combined with information similar to the PERFECT data would be close to ideal for a hip fracture registry.

In conclusion, we found no differences in postoperative mortality after 1 week between hip fracture patients treated with cemented HA and those treated with uncemented HA. Uncemented hemiarthroplasty was found to be associated with more hip reoperations and mechanical complications during the first 90 days after hip fracture surgery.
